# DSC analysis and evaluation of forces released on deactivation of 0.40-mm (0.016") orthodontic thermo-activated NiTi wires: An in vitro study

**DOI:** 10.34172/joddd.2020.002

**Published:** 2020

**Authors:** Vítor Marques Sapata, Diogo Marques Sapata, Julio Araújo Gurgel, Antônio Medina Neto, Adilson Luiz Ramos

**Affiliations:** ^1^Periodontology Department, Universidade de São Paulo, SP, Brazil; ^2^Department of Dentistry, Universidade Estadual Maringá, Maringá, PR, Brazil; ^3^Universidade CEUMA, Dentistry, Sao Luis, Maranhão, Brazil

**Keywords:** Calorimetry, differential scanning, modulus of Elasticity, nickel, Orthodontics, orthodontic wires

## Abstract

***Background.*** This study evaluated the phase transformation of NiTi orthodontic wires and forces they release on deactivation.

***Methods.*** The structural phase transformations of the following five thermo-activated nickel-titanium (NiTi) wires were evaluated using differential scanning calorimetry (DSC): Flexy Thermal Sentalloy® (GAC International), NiTi (35ºC) (Eurodonto), Thermo-Plus® (Morelli), FlexyNiTi® Flexy Thermal (35ºC) (Orthometric) and Damon® CuNiTi (35ºC) (ORMCO Corp.). The wires had a cross-section of 0.40 mm (0.016"). In addition, the forces they released were investigated using the three-point bending test. Five arches of each wire were tested using DSC (-20/80ºC at 10ºC/min), and six arches from each wire were sectioned for bending tests. The data were analyzed with ANOVA and post hoc Tukey tests. Pearson’s correlation test was performed between the results yielded by the DSC tests and those by three-point analyses (P=0.05).

***Results.*** The DSC analysis showed differences between the NiTi alloys from all the manufacturers, with no differences between the lots of the same brand. ORMCO and Orthometric wires exhibited similar TTR values in cooling (P=0.49), and statistically similar TTR values in heating (P=0.056). The three-point bending test showed different patterns in releasing forces. A correlation was found between the DSC analysis and the three-point bending test results.

***Conclusion.*** The higher the temperature transformation was, the larger was the variation of force. All the wires presented higher forces at 3-mm deflection from 155 (±12.3) to 168.1 (±8) cN. The DSC analysis and the three-point bending test showed differences between the NiTi alloys from all the manufacturers.

## Introduction

In the 1970s, Andreasen and Brady^1^ investigated the properties of nickel-titanium (NiTi) orthodontic wires. Due to their high elastic recovery rate and low stiffness, NiTi alloy exerts a constant force suitable for orthodontic movements, and therefore, NiTi wires have been widely used in orthodontic treatments.^2, 3^ Their properties are due to the crystallographic structures that depend on temperature: austenite, martensite, and R-phase.^4^ Low temperatures and high stress favor the martensite phase, whereas the austenite phase is stable at higher temperatures and lower stress.

Many authors have studied the behavior of NiTi wires, in terms of the relations of their properties, shape memory, and phase change.^4-9^

The NiTi wire can be superelastic with or without shape memory or non-superelastic. Superelasticity is a phenomenon in which the wire exhibits a low continuous force with a plateau during a charge or discharge, providing a nearly constant force and offering clinical advantages over non-superelastic NiTi wires.^2,3^ The superelastic wires, or austenitic-active types are capable of undergoing a stress-induced phase transformation from austenite to martensite when loaded or activated.^5^ An orthodontic NiTi wire with shape memory is called martensite-active because it can be deformed in the martensite phase, but when exposed to the oral cavity temperatures a transformation occurs in the austenite phase, allowing the wire to return to its pre-deformed form.^6^

Studies have shown that temperature variations in the oral cavity might be sufficient for inducing repeated phase transformations in orthodontic NiTi wires. Moore et al^7^ monitored the temperatures of central incisors in 20 patients for 24 h and found that the temperature ranges from 5.6ºC to 58.5ºC.^7^ Airoldi et al^8^ found temperature differences between the arches and the teeth when a volunteer consumed cold (5ºC) and hot drinks (60ºC).^8^ Barclay et al^9^ recorded temperatures from 60 volunteers and reported a range of 1.6–65.4ºC.^9^

Differential scanning calorimetry (DSC) is the method used to obtain information about the phases of NiTi wires.^6,9,10^ The three-point bending test was used to determine the elastic deflection of the selected wires and evaluate their superelasticity ability.^11^

This study aimed to use the DSC technique and a mechanical bending test (i.e., the three-point bending test) to evaluate the process of phase transformation of NiTi orthodontic wires and their forces released on deactivation. Accordingly, the following null hypotheses were tested: (1) The behavior in the DSC analysis of the alloys used in wires would not differ between manufacturers; (2) The forces released by different wires would not differ between batches and manufacturers; (3) There would be no correlation between the results of DSC analysis and three-point bending test.

**Figure 1:**
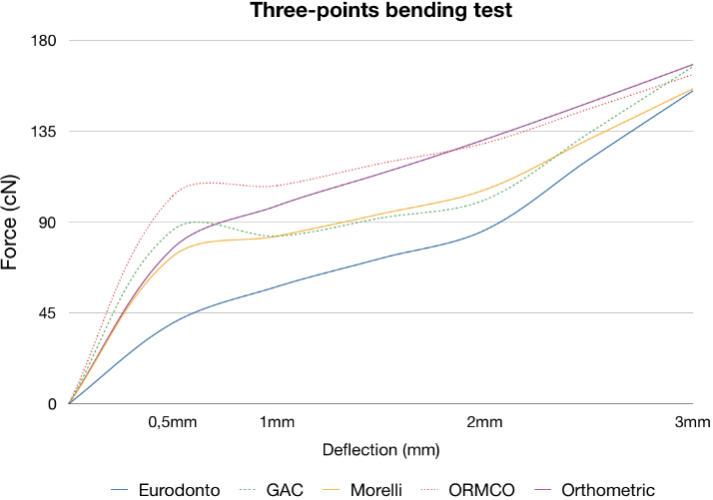
Differences between the tests on the three­point bending test.

## Methods

### Samples

Five brands of orthodontic NiTi wires with cross-sectional dimensions of 0.40 mm (0.016") were selected: Flexy Thermal Sentalloy®e (GAC International), NiTi 35ºC (Eurodonto), Thermo-Plus® (Morelli), FlexyNiTi® Flexy Thermal 35ºC (Orthometric), and Damon® CuNiTi 35ºC (ORMCO Corp.). The manufacturers indicated that the wires were superelastic and thermoactivated at temperatures close to the oral temperature (Table 1).

**Table 1:** Arrangement of materials

**Manufacturers**	**Cross-sectional dimensions**	n
**Flexy Thermal Sentalloy®e (GAC International)**	0.40 mm (0.016")	5
**NiTi 35ºC (Eurodonto)**	0.40 mm (0.016")	5
**Thermo-Plus® (Morelli)**	0.40 mm (0.016")	5
**FlexyNiTi ® Flexy Thermal 35ºC (Orthometric)**	0.40 mm (0.016")	5
**Damon® CuNiTi 35ºC (ORMCO Corp.)**	0.40 mm (0.016")	5

### DSC analysis

We selected 3-mm-long segments. In a previous pilot study, we found no significant difference between wires of the same lot; thus, the first three samples were from the same lot. Two samples from different lots were selected, totaling five samples. The measurements were made alternately and randomly.

DSC Q20 (TA Instruments, Wilmington, Del, USA) was used. Heating and cooling rates of 10ºC/min (TA Instruments, Wilmington, Del, USA) were used. The test sample was first cooled from room temperature to -20°C, warmed to 80°C, cooled to -20°C, and heated again to 80°C, totaling five samples divided into three lots of each wire. The temperature and enthalpy determined by DSC apparatus was calibrated using indium fusion.^10^

The wire segments for each test sample were placed in an aluminum sample holder, and a second empty aluminum sample holder was used as a reference in the DSC test cell. Throughout the analysis, the DSC cell was purged with nitrogen at a rate of 50 mL/minute. The computer Universal Analysis software (TA Instruments) was used to determine the temperature and the enthalpy changes of phase transformations in NiTi wires.

### Three-point bending test

Analysis of the three-point bending test in this study was performed following the ISO 15,841 standard: Dentistry–Wires for use in orthodontics (INTERNATIONAL ORGANIZATION for Standardization, 2006). A universal testing machine was used for the test (DL 1000, EMIC) fitted with a load cell Model S with a capacity of 50 N (5 kgf) and a scanning resolution of 0.01 N (1 gf). An oven was used (Biopar) to perform the tests at the temperature recommended by the ISO specifications.^11^

For the bending test, a device with a distance of 10 mm between the support points supported the wire being tested, and the tested wires were cut into lengths of at least 30 mm taken from the straight portion of the arch-wire. The speed of the tests was 2.0 mm/min, and the forces were measured at the defined displacements at the following deflections: 0.5, 1, 2, and 3 mm, recommended by the ISO. For these tests, the software was programmed to start the test with a deflection of 3.1 mm. The three-point bending test was performed at a constant temperature of 36±1°C. The measurements were made alternately and randomly. Six samples of each orthodontic wire were analyzed.^11^

### Statistical analysis

The data were analyzed by ANOVA and post hoc Tukey tests (force variation). The Pearson correlation test was also performed between the mean values of the results yielded by the DSC analysis and the three-point bending test. The level of statistical significance was set at 5% (P<0.05).

## Results

The results of the DSC analysis and the three-point bending test are presented in Tables 2 and 3, respectively.

**Table 2:** Comparison between the averages, n=5, in DSC analysis, by ANOVA followed by post hoc Tukey tests

	**Eurodonto**	**GAC**	**Morelli**	**Ormco**	**Orthometric**
**DSC (cooling)**					
Exothermic peak (°C)	**29.5**(±1.1) ^a^	**20.2** (±0.6) ^c^	**14.8** (±2.6) ^b^	**-6.3** (±2.4) ^d^	**27.2** (±1.2) ^a^
Ms (onset, °C)	**31.8** (±1.9) ^a^	**22.3**(±0.7) ^c^	**16.4**(±2.5) ^b^	**4.9** (±0.6) ^e^	**35.5** (±2.0) ^d^
Mf (offset, °C)	**27.1** (±0.5) ^a^	**18.1** (±0.6) ^c^	**13.0**(±2.5) ^b^	**-10.6** (±1.1) ^e^	**21.2** (±1.0) ^d^
Heat of transition (J/g)	**3.6**(±0.14) ^a^	**3.5** (±0.19) ^a^	**2.9** (±0.27) ^a^	**9.3** (±1.14) ^c^	**3.8** (±0.41) ^a^
TTR (Ms-Mf, K)	**4.6** (±1.42) ^a^	**4.1** (±0.19) ^a^	**3.4** (±0.07) ^a^	**15.5** (±0.83) ^b^	**14.2** (±2.26) ^b^
**DSC (heating)**					
Endothermic peak (°C)	**33.0**(±1.6) ^a^	**23.6** (±0.7) ^c^	**18.2** (±2.7) ^b^	**15.4**(±1.3) ^b^	**34.0** (±1.8) ^a^
As (onset, °C)	**30.5** (±0.6) ^a^	**21.4** (±0.6) ^c^	**16.7** (±2.7) ^b^	**5.7** (±2.2) ^e^	**26.4** (±1.0) ^d^
Af (offset, °C)	**34.9** (±2.0) ^a^	**25.4** (±0.8) ^c^	**19.9** (±2.7) ^b^	**22.0** (±1.1) ^b, c^	**41.1** (±2.8) ^d^
Heat of transition (J/g)	**3.6** (±0.31) ^a,b,c^	**3.5** (±0.15) ^a,b,c^	**3.3** (±0.30) ^a,b^	**12.8** (±0.44) ^d^	**4.5** (±1.10) ^a,c^
TTR (As-Af, K)	**-4.4** (±1.40) ^c,d^	**-3.9** (±0.18) ^b,c^	**-3.2 (**±0.09) ^b,d^	**-16.3** (±1.23) ^e^	**-14.8** (±3.06) ^f^

*In the same line, different letters represent statistical significance (p <0.05)

**Table 3:** Differences between the means, n=6, the deactivation forces (cN) obtained by the three-point testing by ANOVA followed by post hoc Tukey tests

	**Eurodonto**	**GAC**	**Morelli**	**Ormco**	**Orthometric**
**Force Máx.** **0.5mm (cN)**	**40** (±11)^a,A^	**86** (±11)^b,c,A^	**73** (±18)^b,A^	**103** (±12)^c,A^	**77** (±14)^b,A^
**Force Máx.** **1mm (cN)**	**58**(±7) ^a,A^	**83** (±10)^b,A^	**83**(±9) ^b,A^	**108**(±17) ^c,A^	**97** (±10)^b,c,A^
**Force Máx.** **2mm (cN)**	**87**(±7)^a,B^	**101** (±5)^b,A^	**106**(±8) ^b,B^	**129**(±8) ^c,A^	**130** (±10)^c,B^
**Force Máx.** **3mm (cN)**	**155**(±12) ^a,C^	**167** (±10)^a,B^	**156**(±15) ^a,C^	**164** (±18)^a,B^	**168** (±7)^a,C^

*In the same line, different lowercase letters represent statistical significance (P<0.05); in the same column, different uppercase letters represent statistical significance (P<0.05) (1 cN ≃ 1 g).

The Af (Austenite finish) temperature of ORMCO wire demonstrated similarity with those of the GAC and Morelli wires (P=0.11 and P=0.48, respectively). All the wires differed significantly in As (Austenite start), Ms (Martensite start), Mf (Martensite finish), and exothermic peak (P=0.05). The endothermic peak was similar to those of the Orthometric and Eurodonto wires (P=0.87 and P=0.3, respectively). The ORMCO and Orthometric wires showed similar TTR (transition temperature range) values ​​in cooling (P=0.49) and statistically similar in heating (P=0.056). However, Ms-Mf and As-Af occurred in completely different temperatures between ORMCO and Orthometric (Table 2). The DSC analysis demonstrated that all the wires were significantly different.

The three-point bending test showed that in each group, the force did not significantly improve when subjected to 0.5–1-mm deflection. Forces increased significantly from 1 mm to 2 mm, except for GAC and ORMCO. All the wires exhibited higher forces at 3-mm deflection and released similar forces. However, there were significant variations between the groups at the deflections of .5, 1, and 2 mm (Table 3) (Figure 1).

Table 4 shows the coefficients of Pearson's correlation between the force variations tested and the DSC analysis.

**Table 4:** Pearson’s correlation test between the mean force variations tested for the different wires and their respective DSC results

	**ExP**	**Ms**	**Mf**	**TTR (cooling)**	**HT (cooling)**	**EnP**	**As**	**Af**	**TTR (heating)**	**HT (heating)**
**Force variation among 3 and 0.5mm**	0.89*	0.84	0.90*	-0.48	-0.70	0.83	0.94*	0.65	0.49	-0.72

*Statistically significant (P<0.05)

## Discussion

The 0.16-inch NiTi archwire was used in this research because it is a widely used archwire in daily practice; therefore, the results can be generalized to most arches.

Deflection of the NiTi wires under three-point bending test releases 40–70% less tension when compared with three-point bending tests performed with braces attached (variable according to slot dimension and ligation). Thus, although ISO specifies the conventional three-point bending test as the norm, one should consider these different levels of force before applying them in the clinic. Proportionality is a reasonable method to understand the mechanical behavior of wires. Recently, a similar study was published that compared other brands and sections; however, the correlation between DSC and tension was not tested.^11^

Superelastic capability is attributed in part to a phase transformation from austenite to martensite.^7,12^ This transformation is also induced by stress.^7,12^ Under environmental temperature (i.e., around 21^o^C), Eurodonto and Orthometric NiTi wires were totally martensite (Table 2). During the three-point test (performed at 36±1°C), these wires were induced to transform into austenitic phase, but they still might have had some amount of martensite, as they were under stress; they also had Af close or beyond the test temperature. On the other hand, GAC, Morelli and Ormco, under the three-point test, were expected to be mainly at the austenitic phase during the three-point bending test. Thus, the crystal structure of superelastic NiTi at a given temperature depends on the transformation temperatures, the alloy composition, and the thermo-mechanical treatment of the material,^7,12,13^ as shown by the results of the present study.

Meling and Odegaard^14^ studied the effect of short-term changes in temperature on the torsional stiffness of NiTi wires. They reported that in repeated exposure to cold, the torsional stiffness of some NiTi wires decreased up to 60–85%, whereas a long time was required for the recovery of the initial stiffness. Therefore, the wires with high TTR tend to suffer from a reduction in their stiffness in comparison with those with low TTR. This fact means that low TTR can preserve the austenitic phase even under cooler conditions, maintaining the shape recovery of the wire.

High Af temperatures imply that under oral temperatures, wires are not entirely in the austenite phase, a feature that facilitates the insertion of the wire in the bracket slot. However, it can reduce the capability of shape recovery, thereby reducing clinical efficiency.

The addition of a small amount of metal—such as chromium (Cr), iron (Fe), and aluminum (Al)—to a NiTi alloy, along with an increased amount of Ni, lowers the transformation temperature.^13^ The inclusion of copper in NiTi alloys leads to the greater complexity of the system. In the commercial orthodontic wires studied, copper (Cu) is added mostly at the expense of Ni with a small addition of Cr to lower the final temperature of the austenite phase.^15^ Cu has little effect on lowering the temperature of Af.^16^ Therefore, it is plausible that the proportion of NiTi and/or heat treatment during the production of this wire^13^ leads to the results seen in this study. Cu in the NiTi alloy decreases stress hysteresis, reduces the temperature hysteresis between the formation of austenite to martensite during cooling, and impacts the mechanical properties.^17^

The presence of phase R, which is a transitional phase between austenite and martensite phases, was not observed in any of the tested wires. Brantley et al^17^ theorized that this phase could always be present in NiTi wires when submitted to transformation. However, conventional DSC, compared with the temperature-modulated DSC (TMDSC), is not sensitive enough to detect it in all the cases.

Previous studies^13,18-21^ showed results similar to those presented here for the ORMCO and GAC brands. Despite using NiTi closed coil springs from the GAC brand (Sentalloy), Bawart et al^18^ obtained very similar results for the exothermic (20.6ºC) and endothermic (24.6ºC) peaks. It is noteworthy that Barwart et al^18^ only used one sample in their study.

Iijima et al^19^ evaluated ORMCO (40ºC Cu-NiTi) and GAC (Sentalloy) wires and reported Af of 22ºC for Sentalloy wire and 37–40ºC for Cu-NiTi wire. The Sentalloy wire yielded similar results (25.4ºC), and the 40ºC Cu-NiTi wire yielded different results (22ºC). This difference for the ORMCO wire might be explained by choice of a wire with the highest point of thermal activation (40ºC) that consequently raises the Af temperature.

In 2011, Iijima et al^10^ used NiTi wires from ORMCO (35ºC Cu-NiTi), and the As (7.2ºC to 7.6ºC) and Mf (-14.8ºC to -15.7ºC); the values were similar to those found in this study (5.7ºC and -10.6ºC, respectively). However, five or six samples of the same NiTi archwire were used, in which the possible variation between lots and arches of the same lot could not be determined by the results presented.

Pompei-Reynolds and Kanavakis^20^ also studied the consistency of mechanical properties of Cu-NiTi wires from two different manufacturers in two different lots. They also used the DSC and three-point bending test. Their results showed significant differences between the lots from the same wire manufacturer. They concluded that orthodontic wires of the same material, dimension, and manufacturer but from different lots do not always have similar mechanical properties. However, the authors did not mention how they proceeded with wire randomization for the tests.

Meanwhile, other authors have presented contradictory results to those seen in this study. Berzins and Roberts^21^ observed different values for ORMCO (35ºC Cu-NiTi) and GAC (Sentalloy) wires.

In the DSC test, only the Orthometric wire exhibited the martensite phase at the tested temperature. This means that the heat-activated nomenclature would only be appropriate for this manufacturer because the other wires were in full austenite phases at the time of the three-point bending test. However, there was a limitation in the DSC analysis in this study because the wires were tested without bending.

However, several articles have reported that intrusive or inclination forces should be smaller, given that a merger of this load occurs in tooth apices. Dalstra and Melsen^22^ described that loads between 5 cN and 10 cN would be sufficient for the intrusion of maxillary incisors. During the initial alignment and leveling, a dispersion of the load occurs between the teeth and in an almost incalculable manner when using the straight-wire technique^23^

This variability in behavior between the NiTi wires has previously been reported,^24^ necessitating caution by orthodontists in the choice of the materials used. In the present study, 0.40 mm (0.016”) wire was tested. However, some authors have recommended beginning the alignment and leveling with 0.014” wire.^7,25^ In other calibers, it is noteworthy that the smallest size results in higher deflection and lower load released. An average increase of 0.002” in the cross-section leads to a 50% increase in strength on the plateau, and 0.004” contributes to an increase of up to 150%.^26^

Abdelrahman et al^27^ studied the effectiveness of three settings of NiTi arches (3M – Unitek TM): conventional, superelastic, and thermoelastic, 0014", and found no difference in the efficiency of wires during the alignment phase. However, wires from only one manufacturer were tested.

On heating, there was a significant positive correlation between As and the variation of force (Table 4). It was found that the greater the variation of force, the higher the temperature required for initiating the formation of the austenite phase in the wire, which also resulted in a considerable variation in the plateau (Table 4).

Although it was not statistically significant, with a lower correlation, the TTR - for both cooling and heating - was negatively related to the force variation. Therefore, the higher the TTR, the lower the variation in strength. This behavior was clearly seen in the ORMCO and Orthometric wires.

A similar study^28^ evaluated the temperature transition in thermal rectangular 0.019" × 0.025" NiTi wires, using the DSC analysis. The brands GAC and Highland Metals exhibited thermal characteristics, but Aditek, Morelli, and Orthosource did not. The wires of Ormco and Orthometric brands exhibited martensite phase during a longer period, promoting smaller loads of force in the oral environment.

Another clear, yet insignificant correlation was HT, where the ORMCO wire obtained the highest levels of force (up to 100 cN) and showed significantly higher need of energy than those of all the tested wires to change phase (9.29J/cN on cooling and 12.76J/cN on heating). In other words, the more energy required to change the phase, the greater the response of the wire, and therefore, the greater the force released.

With the results obtained in this study, it is still necessary to investigate the rectangular NiTi wires, include aging in the oral medium, and compare the results of the wires before and after this aging, taking into account the effect of biofilm.

## Conclusion

- The DSC analysis showed differences between the NiTi alloys from every manufacturer, with no differences between the lots of the same brand.
- The three-point bending test showed different patterns in releasing forces, and a correlation between the results of DSC analysis and the three-point bending test.
- The orthodontist should be aware that the 0.40 mm (0.016") NiTi alloy has a wide range of properties and levels of forces between brands.

## Acknowledgments

None.

## Authors’ Contributions

The VMS wrote and conducted the article, the DMS submitted de article, the JAG and AMN contributed to the discussion section, the ALR analyzed and interpreted the data and guided the article and the authors. All authors have read and approved the final manuscript.

## Funding

This study was conducted with the resources of the authors.

## Ethical Approval

Not applicable.

## Competing Interests

The authors declare that they have no competing interests.
